# XR Programmers Give Their Perspective on How XR Technology can be Effectively Utilised in High-Performance Sport

**DOI:** 10.1186/s40798-023-00593-5

**Published:** 2023-06-13

**Authors:** Peter J. Le Noury, Remco C. Polman, Michael A. Maloney, Adam D. Gorman

**Affiliations:** 1 School of Exercise and Nutrition Sciences, Faculty of Health, Queensland University of Technology, Brisbane, QLD Australia; 2 Institute of Health and Wellbeing, Federation University, Berwick, VIC Australia; 3 Australian Institute of Sport, Canberra, Australia

**Keywords:** Motor skill, Perceptual–cognitive skill, Virtual reality, Representative design

## Abstract

**Background:**

The successful use of extended reality (XR) in sport is highly dependent on the extent to which it can represent the perception–action couplings that exist in the performance setting. However, there are many unknowns regarding the effectiveness of XR technology which is limiting its adoption in sport. Therefore, providing high-performance sporting organisations with more information about the efficacy and utility of XR, specifically its strengths and limitations, is warranted.

**Results:**

The results provide insight into the limitations of XR and how those limitations are likely to reduce the effectiveness of XR for training motor skills. The participants described opportunities provided by XR for measuring athlete performance and highlighted several practical applications for enhancing athlete and coaching performance. Using artificial intelligence (AI) for training tactical decision-making and creating new movement solutions was also a key finding.

**Conclusions:**

The use of XR in sport is in its infancy, and more research is required to establish a deeper understanding of its utility and efficacy. This research provides sporting organisations, coaches, athletes, and XR technology companies with insights into where XR technology can have the greatest positive impact on performance in sport.

## Background

Identifying novel methods to enhance athlete performance is a strong interest of practitioners working in high-performance sport. As a result of some promising results from training interventions in other domains such as psychology [1], medicine [2], and the military [3], there has recently been a proliferation of interest in how extended reality (XR) technology can be applied to sport. XR is defined as an umbrella term that encapsulates all real and virtual environments generated by computer technology and wearables and includes virtual reality (VR), augmented reality (AR), and mixed reality (MR) [4] (for a detailed review, see [5, 6]). Whilst XR technology has been used in sport to successfully train perceptual–cognitive skills such as decision-making, pattern recognition, and understanding of team tactics (tactical decision-making) [7, 8], there is evidence to suggest that there may be limitations in the use of XR technology when training motor skills [9]. Improvements in research design (e.g. inclusion of adequate transfer tests, control conditions, and comparisons to representative practice tasks) are required to provide more clarity on the efficacy of XR tools and the extent to which they can enhance performance in real-world settings [5].

An important theoretical principle for assessing the efficacy of XR technology is representative design which considers the extent to which a training modality accurately represents the perception–action couplings that exist in the performance environment [10]. The number of sport-specific XR tools that have been formally assessed for how well they represent the real performance setting is relatively small; however, exceptions include investigations into animated VR in tennis [11] and baseball [12] and an AR indoor volleyball simulation [13]. The results from these studies found no significant difference between actions performed in the XR environments and real-world settings, suggesting that athletes could judge or interact with the trajectory of the balls in tennis [11], baseball [12], and volleyball [13] in a similar manner in both XR and real-world conditions. Whilst these studies provide some evidence that the information athletes were using to inform their actions may have been representative, further research is required to investigate the representativeness of other behaviours in the XR environment such as the timing of interceptive actions and the nature of the haptic information that is available [5]. Given the level of representativeness of XR tools is an essential factor in whether skill transfer occurs to the performance setting, it is essential to understand the extent to which XR is capable of simulating the performance environment [5] (see also [10]). Indeed, the lack of research in this area and the high number of unknowns around the efficacy of XR technology in sport is a barrier that is limiting sporting organisations from adopting this technology.

Key to resolving this issue is providing high-performance organisations with more knowledge and understanding of XR technology, including the different forms of XR technology, their capabilities or limitations, and how the technology can best be utilised in high-performance settings. Indeed, many practitioners have creative ideas of how XR tools could be used to train skills such as anticipation or performance under pressure; however, they often do not have the level of expertise required in the area of XR technology to confirm whether these ideas can be developed and implemented effectively. A key step towards improving practitioners’ understanding of how XR can be utilised is to gain knowledge from XR programmers who are experts in writing code and programming the software required to develop various XR tools. XR programmers can help practitioners determine whether their XR training ideas are possible to achieve, the limitations associated with XR tools which may influence training effectiveness, and the type of data required (e.g. Vicon data) to create a highly representative XR tool. To our knowledge, no study exists in the literature that has collected data from XR programmers (the experts in this field) to help inform sports practitioners about the potential of XR to be applied in sport performance programmes.

Therefore, the aim of this study was to interview XR programmers about the potential of XR tools for application in high-performance sport. Given that maintaining task representativeness is considered to be crucial to facilitate the transfer of perceptual–motor skills from the training environment to the performance setting [10, 14], the interview questions focused on the potential of XR tools to simulate a real-world competition environment, including aspects such as the look of the environment, the functionality of objects, and the ability to replicate individual player kinematics and the movement patterns of teams. The interview questions also focused on performance measures and how they can be collected in the XR environment, as well as the utility of XR and how it could be used to train athletes’ skills. Although this study was exploratory in nature, the aim was to provide practitioners with an in-depth understanding of the extent to which sport-specific situations can be simulated using XR tools, the types of information that can be presented to athletes using XR (e.g. movement patterns of a defensive team structure or offensive play), and the potential to simulate representative actions when interacting with virtual objects, thereby providing guidelines for the utilisation of XR technology into the future.

## Methods

### Participants

XR programmers (*n* = 7) were recruited from various companies that specialised in VR, AR, and MR development. Given that it was essential for participants to have a thorough knowledge of the use of XR technology in sport (a rare topic of specialisation), purposive sampling was used in this study to help select participants. The research team used their pre-existing links with XR programmers to contact programmers via phone or email to ask whether they would like to be involved in the research. Subsequent phone calls or in-person meetings were organised if participants had any further questions. The pool of potential participants who were contacted were based in Australia, the UK, and the USA, but the final sample included participants from Australia and the UK only. The XR programmers had expertise in developing the software and hardware required to create animated XR simulations (including animated VR, AR, and MR simulations) that allowed users to interact with a virtual environment or objects. Their skills also included excellent knowledge of Unity (a platform that enables the development of computer games and other interactive 3D graphics applications), and an in-depth understanding of various computer programming languages. Participants were required to have a minimum of 2-year experience working professionally as an XR programmer (or equivalent position) to participate in this study, and participants were required to have been involved in at least one sports-related XR project. The small sample size reflects the rareness of this group within the broader community around the world, and the small portion of XR programmers who have experience working on sports-related projects (although the number of these projects is growing each year). The participants provided informed consent, and the study received institutional ethics approval. The COREQ guidelines have been followed in the reporting of this study [15].

### Data Collection

Semi-structured interviews were conducted either face-to-face at the participants’ workplace or via Zoom by the first author who was trained in qualitative research and had over 4-year experience working with XR programmers. Moreover, the first author had a strong interest and understanding of XR technology and how it can be applied to high-performance sport. A relationship was established between the first author and the participants prior to data collection, which was based on communication with participants about the study objectives, as well as understanding the participants’ background and suitability for being involved in the study. The first author and participant being interviewed were the only people present during the interviews.

The interview questions were developed by all four authors which included an expert in the field of qualitative research. The interview questions were divided into two main sections including a section on the participants’ background, and a section focused on the representativeness of XR for simulating the real performance setting and how XR can be utilised within high-performance sport. Probing questions were used throughout each participant’s interview to further elaborate upon the ideas that were expressed and to ensure the participant’s descriptions were accurately understood by the interviewer [15]. This approach ensured participants’ responses to each question had consistent depth and complexity and gave participants flexibility to respond in ways that were outside the scope of the specific question [16]. Pilot interviews were conducted with two XR programmers to examine the appropriateness of the questions and overall flow of the interview [17]. This helped the interviewer to understand the XR programmers’ formal and informal language and to develop suitable probe questions. This process resulted in questions being removed and the wording of some questions being changed, and so, the pilot data were not used in results section of this study.

The interviews were audio recorded using a smart phone device, and the duration of the interviews ranged between 32 and 58 min (*M* = 40.5 min, *SD* = 10.05 min). The interviews were transcribed using the Otter.ai program and were analysed using NVivo 11 software (QSR International Pty, Ltd., 2017). Notably, participants were recruited for this study until no new relevant knowledge was being obtained from new participants (i.e. data saturation).

### Data Analysis

The data analysis process followed a six-phase thematic analysis approach which was based upon the recommendations of Braun and Clark^18^. Although this approach will be described in a linear fashion below, it was actually an iterative and reflective process that developed throughout the analysis and involved continuous back and forth movement between phases. *Phase one* involved the first author familiarising himself with the data by listening to the audio recordings of each interview, thoroughly reading the final transcripts, and documenting any theoretical and reflective thoughts that developed throughout the data immersion process, including interests and growing insights about the research topic [19]. *Phase two* involved generating initial codes in the data. This included the first author systematically working through the entire data set, paying close attention to each data item, and identifying any interesting aspects of the data that may have formed the basis of themes. Important sections of text were highlighted and attached with labels to index them as they related to a theme or issue in the data. *Phase three* involved searching for themes which began when all data had been initially coded and organised, and a list of different codes identified across the data set had been established. This phase required sorting and collating the potentially relevant coded data into main themes and subthemes [20]. Thematic networks were used to create a web-like network to organise codes and themes. Any codes that did not seem to fit with any particular theme were categorised as “miscellaneous” [20]. The *fourth phase* involved reviewing the themes generated. The first author reviewed the coded data extracts for each theme and considered whether they appeared to form a pattern. The validity of each theme was deliberated to determine whether it accurately reflected the meanings evident in the overall data set [20]. During the *fifth phase*, the first author defined and named the themes generated. This involved establishing what feature of the data each theme captured and identifying what was of interest about those themes and why [20]. The first author conducted and wrote an in-depth analysis about the story each theme was telling and deliberated with the research team throughout this process [20]. *Phase six* involved writing a report of the thematic analysis. Short quotes or extensive passages were included in the report, where appropriate, to aid the reader’s understanding of specific points of interpretation and to illustrate the prevalence of themes. All relevant results are discussed in results and discussion, including unexpected results, or results that did not correspond to the main issue being studied [21].

### Research Quality and Rigour

To enhance the quality of this research, the first author used the process of reflexivity and engaged in conversations with “critical friends” [19, 22]. The research team involved in this study acted as critical friends and encouraged the first author to reflect on the interpretation of data and to question the data analysis method [23].

## Results

**Fig. 1:**
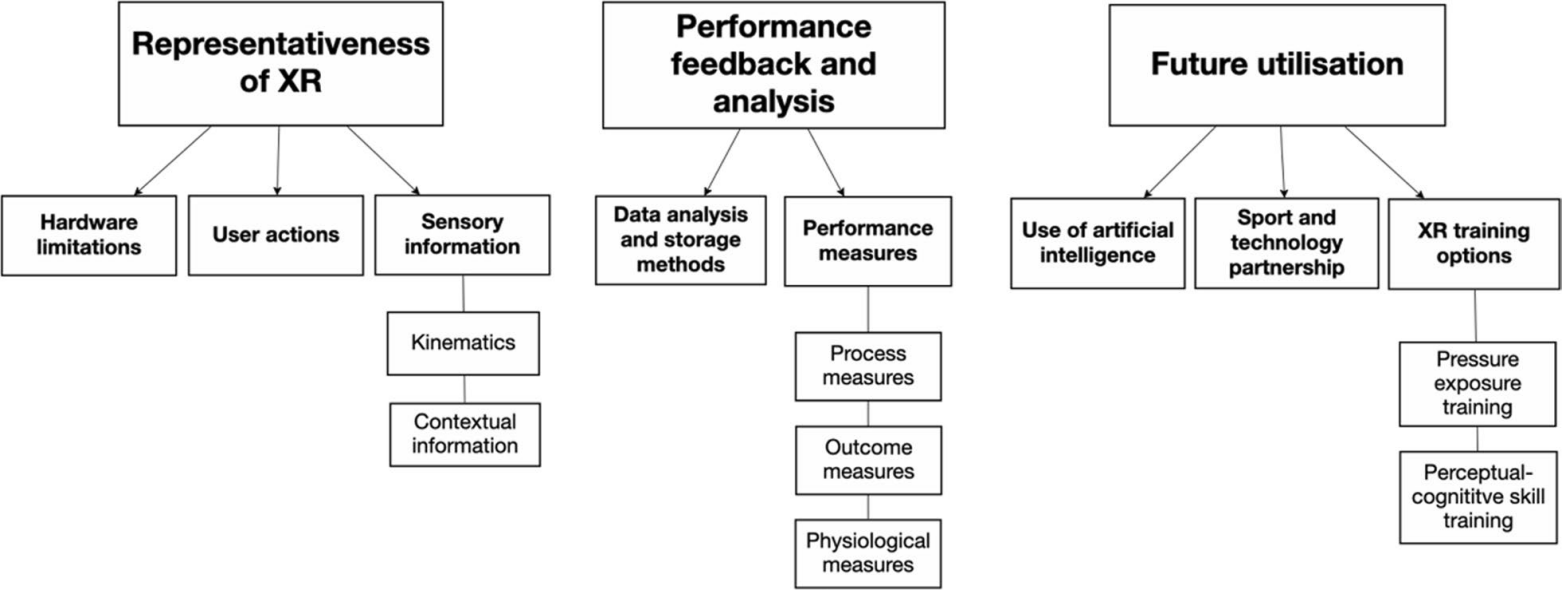
Higher-order, second-order, and third-order themes generated from the interview data

Three higher-order themes were identified during the thematic analysis including (1) *representativeness of XR,* (2) *performance feedback and analysis*, and (3) *future utilisation* (see Fig. 1). Supporting subthemes linked to these higher-order themes were also identified and are illustrated using representative quotes from the XR programmers where applicable. To protect the identity of participants and maintain confidentiality, all participants were assigned a pseudonym label (e.g. P1–P7).

### Representativeness of XR

The representativeness of XR technology (the extent to which the XR environment replicates the real performance setting) [5, 10] was a higher-order theme that emerged from the interviews with the XR programmers. This theme was described by the XR programmers by (a) addressing a number of limitations associated with XR hardware including VR and AR headsets, (b) considering the representativeness of user actions and the outcome of those actions when performing in the XR environment compared with the real-world performance setting, and (c) discussing the representativeness of sensory information presented in XR environments including kinematic information (e.g. the movement of a virtual opponent) and other forms of information such as the trajectory of the ball for a tennis shot or crowd noise.

#### Hardware Limitations

The XR programmers described how the representativeness of XR tools can be affected by hardware limitations. Specifically, VR headsets can have negative effects on user’s visual capabilities when inside virtual environments. Examples of these negative effects were mentioned, including having difficulties with seeing objects that are far away, underestimating the depth of objects, and the resolution of visuals being different to that experienced by the user in the real world.

P7: “In [some sports], in terms of distance, it’s really hard to see the other person and that's something that's currently a bit of a limitation around perception.”

A significant limitation that was noted by the XR programmers included the limited frame rate and/or refresh rate of VR headsets which they believed would change the timing of actions in an animated VR environment compared with the timings used in the real-world setting. An example of playing tennis in an animated VR environment was provided to support this statement. It was suggested that the timing of swinging a tennis racquet or arm to contact a virtual object such as a tennis ball is not representative of real-world performance.

P2: “the user might see the ball coming in frame-by-frame, and if you were to do it in slow motion there would be a gap between the balls…And I've timed my swing for somewhere in the middle of that. So, there's a little bit of wiggle room there.”

A number of limitations associated with the use of AR glasses were mentioned. These limitations included a limited field of view (30-degree field of view diagonally across) which means that the viewer only sees a small window of visual information, requiring the user to swivel their head to see the bigger picture. The brightness of objects was also described as being poor which they felt would decrease the realism of those objects. The programmers estimated that it could take 10 years of further development before AR technology would be capable of presenting visual information with a level of realism that is comparable to that seen in the real-world environment.

#### User Actions

The representativeness of user actions when performing in the XR environment was described as being low by the XR programmers. This was noted as being due to the lack of realistic haptic feedback (touch feedback) that is present when users perform motor skills. Additionally, it was highlighted that athlete movements and body positioning when attempting to perform actions in the animated VR environment are not representative of the real-world setting. It was proposed by the programmers that there is no value in performing actions in the animated VR environment that rely heavily on haptic feedback. In fact, it was believed that doing so could be detrimental to performance.

P5: “I find when people try to take a touch in VR football, they take a really artificial body posture. So, they like lean over the ball as they do it, because they're almost trying to track it as much with their eyes as possible, which means they're sort of losing that proprioceptive feedback that they use in real life. So, they end up…really arching their back over the ball to take a touch, which is just not natural.”

In addition to user actions not being representative of the real-world setting, the programmers expressed that the interactions that occur between the user and virtual objects are not representative of the real world. This was thought to be caused by both the lack of haptic feedback and poor responsiveness of tools used to perform actions (e.g. the force on a bow when using the arm and hand to draw the bow in archery, or a virtual tennis racquet producing the desired spin and force on a virtual tennis ball), as well as the inability to accurately simulate the physics of objects as they move through space after the user has interacted with those objects. It was stated that athletes who have completed many hours of training in the real-world setting can notice the difference when performing those skills in the animated VR environment.

However, the XR programmers discussed that in some sports user actions performed in the animated VR environment can be highly representative of the real-world setting. It was described that physically interacting with real-world objects (instead of virtual objects) when using an animated VR tool allows haptic feedback and the responsiveness of equipment to be maintained whilst still looking at animated visual information (e.g. a quarterback throwing a real football that is portrayed or seen as an animated football when viewed in the VR environment).

Finally, and as mentioned in the hardware limitations section, the timing of actions when performing in the animated VR environment was not considered by the programmers to be representative of the real-world setting. They stated that this was caused by the slow frame rate and/or refresh rate of VR headsets.

#### Sensory Information

The XR programmers described the representativeness of sensory information presented in the XR environment by discussing kinematic information (i.e. the movement of opponents) and contextual information (e.g. crowd noise or how a virtual baseball moves through the air).

##### Kinematics

It was reported that kinematic information can be presented within XR environments with high representativeness. The XR programmers described that this is possible through the use of motion capture systems such as Vicon. Athletes’ movements could be captured and transferred into the animated VR environment to provide the user with representative information to help facilitate performance (e.g. anticipation of an opponent’s actions). It was highlighted that investing the necessary time and money into a comprehensive method of motion capture at the start of a project can result in a more representative final product that allows for more to be done in terms of developing the simulation in the long term (i.e. greater scope for customisation). In addition to the use of Vicon for capturing motion, it was noted that it is also possible to extract kinematic information of athletes from historical 2D video footage.

P7: “Technology is getting really good at looking at two dimensional [video] of a human and extracting sculptural data from it. Anyone using Snapchat filters, or something like that, sort of seeing how we can look at a face and sort of work stuff out. So that sort of technology is starting to get to the point where we may be able to start pulling this data from data sets that haven't been specifically set up for it.”

However, the programmers mentioned a limitation of using Vicon or 2D video footage to capture the motion of athletes, which is that these methods may not be able to capture information about where an athlete is looking at a given time during their motion (i.e. it is unable to capture eye movements). Indeed, it was described that if information that assists performance is lacking in the animated VR environment during training (e.g. information about where an opponent is looking), this means that athletes are not basing their actions on information that is comparable to that of the real performance environment which, in turn, could have a potentially detrimental impact upon skill acquisition.

P7: “which way the person's looking… Maybe in rugby, that's really important. And who knows, maybe…because you have to do the little thing where they're like, I'm gonna throw the ball, whoa [throwing a dummy pass]….maybe the really top end players might not even realise it, but they're actually looking not just at the ball, but the person's eyes to see that they're actually trying to look for the gap. We could accidentally anti train by not using really good Vicon with facial capture.”

##### Contextual Information

The XR programmers identified that contextual information such as the appearance of physical objects, including the features of a specific sports stadium and the presence and noise of the crowd, can all be presented with high representativeness in a VR environment. Other features mentioned that could be simulated included specific weather conditions (e.g. humidity, altitude, and various temperatures) and different playing surfaces, both of which affect the way that animated objects behave in the environment (e.g. the way a tennis ball bounces off a clay court versus a grass court). However, whilst these contextual features can be simulated in the animated environment, it was expressed that it can be challenging to create XR tools that maintain high levels of representativeness of the behaviour of physical objects after those objects have interacted in some way with those contextual features. For example, it is challenging to simulate (with high representativeness) the trajectory of a tennis ball as it moves through the air when interacting with wind in an animated VR environment. Nevertheless, the XR programmers suggested a solution to this problem which involved capturing the dynamic changes of wind and how it affects physical objects live during competition (e.g. the trajectory of tennis shots over the course of a tennis match), and then importing this information into the animated VR environment and replaying it.

### Performance Feedback and Analysis

The XR programmers discussed many ways in which athlete behaviour can be measured, analysed, and then stored during and after using XR tools. Specifically, data analysis and storage methods were a subtheme that emerged throughout the interviews. Moreover, the XR programmers mentioned various performance measures that can be captured in the XR environment which were divided into three further subthemes including process measures, outcome measures, and physiological measures.

#### Data Analysis and Storage Methods

It was stated that the performance of athletes in the animated VR environment can be analysed and this information can then be broken down, stored, and presented in any way that coaches or players wanted. An example provided by the programmers included automatically tracking decision-making and attributing values to different decisions that were made, thereby providing athletes and coaches with a better understanding of decision-making behaviour.

P3: “If I pick option A, that gives me plus one to respect and integrity, if I pick option B that gives me plus one to individualism or wanting to win. Any kind of value you want to attribute to a decision, you can tally that up. And then you can show people at the end, kind of okay this is the path you took”.

The programmers described how the data captured in the VR environment can be broken down into a customised format, providing coaches with more specific information and insight into athlete performance.

P4: “So was it a shot with spin, was it a shot with high power? We also calculate the highest difficulty. So, we divide the goal into zones, and then we take the ball speed, spin height, etc., and then we imply a level of difficulty for each shot. So, the goalkeeper coach has a reference for was that a difficult shot or an easy one”.

P4: “So say, for example, you have 20 shots, and you save 19 of them, you probably want to know what's going on for that one specific shot. So, we can click on that chart, and I'll give you a detailed breakdown of all the different types of characteristics of what that goalkeeper was doing. And then you'll go, okay this all kind of makes sense, but maybe you just hesitated a wee bit too long”.

The XR programmers discussed how athlete behaviour in the animated VR environment can be stored by recording athletes’ every move via a third person perspective camera which exists in the VR space (the camera essentially follows the athlete in the VR environment without interfering with their behaviour). The resultant video could then be used by coaches and athletes to analyse performance. Another way to use video to analyse performance that was mentioned by the XR programmers was to record behaviour from the athlete’s viewing perspective. They described that behaviour could be recorded from this perspective in specific situations (e.g. recording 5 s of lead in time to when an opponent has a shot on goal in a soccer goalkeeping simulation) and this could be stored and watched by coaches. It was noted that the data captured whilst performing in the VR environment can be presented using a number of presentation methods to provide coaches and athletes with more efficient ways to analyse performance (e.g. on a website, using bar charts or other graphs, and schematic diagrams). These data could also be presented using these methods live during competition or training.

#### Performance Measures

The XR programmers discussed the potential to measure various components of athlete behaviour when performing in the XR environment. These measures were divided into subthemes including process measures, outcome measures, and physiological measures.

##### Process Measures

Gaze behaviour was identified as a measure that can be captured by the XR tool to assess athletes’ visual scanning behaviours and also identify the specific objects that are attracting the athletes’ visual focus.

P2: “We can apply tracking [eye tracking behaviour] to see what exactly their eyes are focusing on when they're on the pitch or out on the field. What is it that they're scanning? What are they focusing on? Are they looking off into the crowd getting distracted? Are they focusing on the ball? Are they noticing all the people around them?”

Although gaze behaviour was suggested to be a simple measure to record when using animated VR, the XR programmers pointed out that it is more difficult to measure depth of gaze behaviour. It was described how gaze behaviour tools will capture what the athlete is seeing, but as soon as the gaze behaviour hits a solid object in the environment, it will select that as the athlete’s focal point. For example, in sports involving a net (e.g. volleyball), gaze behaviour tools may identify that the athlete’s focus is on the net, when their focus is in fact on the players standing beyond the net. Nevertheless, according to the programmers, there could potentially be new methods of measuring gaze behaviour in the animated VR environment. For example, because animated VR is a three-dimensional environment, XR programmers could measure where athletes are looking volumetrically (in the volume of the playing space) to infer depth of gaze. Heat maps could also be used to display volumetric gaze behaviour data across specific moments in time.

P7: “So, I know that there are 16 other footballers and a ball. I could show not just volumetrically, but per person per object per thing that is important, where the dwell times are and when the dwell times are. And that's something that is really hard to get. So, you can get standard volumetric, and also like per categorised object.”

The XR programmers mentioned that issues can arise when measuring gaze behaviour, particularly when athletes frequently and rigorously move their head. However, it was suggested that the hardware is progressively improving, and so, the programmers suggested that it was important to select the appropriate hardware to measure gaze behaviour.

Facial movement was another component of behaviour that was described as something that can be measured when using animated VR via the use of headsets that include built-in skin conductance cameras to track facial movements (e.g. mouth movements). Moreover, according to the programmers, it is possible to measure the movements of athletes as they are performing in the animated VR environment using VR specific tracker devices (e.g. VR headset, Vive tracker) that are placed on the athlete’s body. However, it was noted that the accuracy of these measures would not be to the level of motion capture measurement systems such as Vicon which can support 64 reflective markers, compared with approximately 16 tracker devices in the VR setting.

P3: “In theory you can get things like position and direction of their head, hands, and anything else you attached a tracker to. You’ll need sensors on any part of the body you want specific data for.”

##### Outcome Measures

Outcome measures that can be captured in the animated VR environment were discussed, including response time via the use of tracking devices, and the behaviour of virtual objects. For example, the programmers described that it is possible to measure the speed of a returning shot in tennis and the speed at which a batter swings their bat in cricket.

P6: [on measuring response time] Absolutely it can yeah like the same sensors and a headset are even better actually than what are in phones so there's a gyroscope there's an accelerometer, but it's also got vision-based sensors as well. So, you just use essentially sensors to say when the headset started moving this direction, this way, this time to the millisecond and get the timestamp.”

Decision-making was also described as a variable that could be measured in the VR environment to help inform recruitment decisions.

P2: “Now you're going to scout this player because he did well on the quiz [decision-making task]. And it could be a good way to sort of prune out some of the choices…For like more static responses, like a player should know, if you're in this position, you'd give it to that, it's like the perfect spot. If they're not choosing that, then maybe they're not going to be right for that recruitment goal there.”

Interestingly, the XR programmers described that for VR tools that require an autonomous system (e.g. a human competing against virtual basketball opponents in a VR system), the decision-making of the autonomous system can be measured and recorded. They believed that this could provide information to coaches about why the system chose to make particular decisions in various situations. Autonomous systems are programmed much like a video game, that is, they make decisions that comply with the rules of the game, but the programming is ultimately designed to give the system an advantage or help it to win. Therefore, analysing the decision-making of the autonomous system may provide coaches and athletes with a different perspective on how to gain an advantage.

##### Physiological Measures

Many physiological measures that can be recorded in the animated VR environment were identified by the programmers. Essentially, they reported that any of the physiological measures that can be captured in the real-world can also be captured in the VR environment. Examples included heart rate and galvanic skin monitors to measure physiological responses to stimuli and EEG to measure an athlete’s cognitive state.

### Future Utilisation

The future utilisation of XR technology in sport was a primary theme discussed. Stemming from this overarching theme, the XR programmers discussed how artificial intelligence (AI) can be utilised in sport in the future, the importance of developing strong partnerships between people working in sport and people working with XR technology, and how XR technology can be utilised to enhance athletes’ decision-making skill and performance under pressure.

#### Use of Artificial Intelligence (AI)

Machine learning techniques that use AI were reported by the programmers as having a high potential for positive impact on sport by assisting with tracking the motion of athletes during live competition. They described how athletes’ movements can be tracked at a basic level (i.e. where players move in space more broadly) by using conventional video cameras; however, this method would not necessarily provide precise kinematic data of individual athletes. Alternatively, it was suggested that a camera called ‘event camera’ could be used for tracking athlete movements, which is capable of giving coaches a more precise idea of player motion in a given space.

P3: “There's another type of camera now…called event cameras…It only captures data on movement. So, if a pixel was different to what it was a millisecond ago, or whatever time period it's capturing, it'll record that as an event. So rather than getting 60 frames per second of a full image, you're seeing all these dots of movements, and you end up with a very precise kind of idea of the motion happening in the scene.”

AI was mentioned as having high potential for use in identifying and categorising specific types of athlete movements in specific performance environments. The programmers described how experts in the sport of interest could watch past footage of a game and categorise the different movements or skills being performed. They stated that the AI, through the use of learning-based algorithms, could then learn from these categorisations and start to automatically categorise movements and situations from all previous games played. For example, coaches could use historical data of a particular team to inform the creation of playing tactics.

P7: Okay, cool, here's all the [specific team’s] games that have ever been played. Now we have a database to categorise stuff because we're starting to get [to] the point with AI where we can start doing auto categorisation and learning algorithms…you can start doing really fancy statistical modelling on this and start saying, well, when someone does this, 90% of the time this happens. But this could happen, like you could actually start getting statistics from it as well as just teaching people.”

The programmers highlighted generational adversarial neural networks (GAN) as a type of AI that can be used to categorise historical data and importantly, produce new data. They described how AI systems can be developed in such a way that they are capable of generating their own Vicon data and can essentially simulate how a person would behave in different situations. Notably, they believed that once an AI system has enough information (e.g. enough movements and situations are categorised), it can start to produce its own solutions to problems that potentially no other human has ever previously considered. An example was given of how GAN can be applied to tennis.

P7: “You also could then go through and just auto categorise every tennis game that's ever been recorded. And use that data to start doing some really funky analysis. And at the same time, you could use the other side of that system to start producing really good tennis simulators, and possibly even come up with new shots or angles because at that point, you can start really letting the computers crunch things and be like, okay, cool, we now know all the shots and you now know what the percentages are…If somebody comes in this way, what's the shot that you generate? And you're like, well, we didn't think about that, did we?

AI technology was described as very useful for identifying the current state of play and anticipating what will happen next. This could be beneficial during training; however, it could be particularly helpful for coaches during live competition to help them to inform their athletes about tactics.

P7: But it's one of the things that AI is really good at, which is what comes next. Give it enough information it can predict the future…you can end up with a system that works out where everyone is, and the system goes, they're going to do this on this play. Now imagine as a coach…really good just to be like instantly know within a pretty large certainty what's going to happen. Sure, you can use that in training, but you can use that live.

#### Sport and Technology Partnership

The programmers described the importance of collaborating closely with coaches and athletes when designing purpose-built XR tools. Discussing the problems that sports are facing and having strong communication with coaches to collaboratively develop solutions was believed to be an important step in the process of developing XR tools. Understanding from the onset of development exactly what needs to be in the XR tool and what variables should be changed or customised over time is essential information that the XR programmers needed to help them to develop a practical and sustainable XR tool.

P7: “Oh, very important [partnership between sport and XR programmers], because…we understand the systems…that we're using, but without working really closely, especially moving further into the realms of high performance where things are built on margins… it becomes really important to be able to have a closely coupled…development environment where you have a lot of back and forth.”

#### XR Training Options

The programmers provided insights into how XR technology can be used to enhance skills in sport. They described how animated VR could be used for exposing athletes to pressure or high stress environments, as well as for developing perceptual–cognitive skills such as tactical decision-making and anticipation.

##### Pressure Exposure Training

Exposing athletes to highly representative competition environments through the use of animated VR is a training option that was believed to have the potential for positive outcomes. The XR programmers expressed that the anxiety and nerves associated with competing at high stakes events such as the Olympic Games can be overwhelming, particularly for younger athletes who are less experienced. Therefore, exposure to these environments using animated VR in controlled circumstances and with appropriate support was suggested as being potentially useful for helping athletes to regulate their emotions. Indeed, the programmers stated that one of the easiest aspects to develop for an animated VR simulation is the sensory information in the environment such as the presence of a crowd or the features of a stadium. They believed that the results from exposure training could help identify possible problems for a given athlete which is information that could subsequently be used by the XR programmers to help develop a solution.

P3: “If you need someone to feel what it's like or experience what something is like, then VR is probably the way to go, it does give you that more immersive, experiential kind of exposure.”

P7: “All the lights, all the cameras, all the booing the yelling… and just see how their response goes…and just see whether…we're getting a really strong reaction…. And then it would be getting the response and the conversations that have been linked from that…I'd start looking at where the problem space is.”

##### Perceptual–Cognitive Skill Training

Training athlete’s tactical decision-making and anticipation skill using animated VR and AR was mentioned as an effective way to train an athlete’s ability to identify kinematic information that can help to inform their anticipation.

P5: “[A cricket VR simulator is] brilliant for picking up cues from the bowlers and sort of like coming out of the hand, stuff like that, because you've got very good hand tracking technologies… and people use that in collaboration with motion capture suits, which means you can capture the full body, including the fingers, which means you can get those complexity of releases.”

Furthermore, the programmers highlighted how tactical decision-making training can be developed using animated VR by presenting animated scenes of competition conditions including the physical movements or patterns of play of opposition teams (e.g. the ball movement of a team in Australian rules football). Notably, they described how all players in a team could be in the same VR environment at the same time and be able to interact with that environment to solve problems or have discussions about the tactical information that they are experiencing.

P3: “If you want people to see… the tactics or play planning in VR… they could jump in and have like, first-person perspectives of it. You could get multiple people in the same scene, all observing an example, or observing one situation. That kind of thing gets really cool, because if you’re in a joint experience, we could also see each other and wave around and point to things and I'll say to you, oh, yeah, see where this winger is? And you go, Oh, yeah, I can see him- draw a circle.”

However, the programmers cautioned that there are weaknesses associated with this kind of tactical decision-making training using animated VR. That is, the movement kinematics of players in the environment would not be specific: The movements would simply be a generic animation of running or kicking technique which could be sufficient, depending on the aim of the training session.

Lastly, AR was mentioned as a tool to use for educating athletes on different concepts (e.g. offensive or defensive tactics). AR was described as being an efficient way of delivering information to athletes in an immersive way (which has been a weakness of past information delivery systems used in sport, including the use of Power Point presentations or white boards^5^).

P3: “AR is a really accessible way of delivering information to someone quite quickly, in a really unique and immersive way. So, you can look at like, okay, here's an example of human anatomy and the parts of your body that have been impacted negatively by taking these substances [drugs]. So, …. you're hearing information being narrated, you can read it, you can see visually kind of what's happening at the same time. So, as an educational medium, I think it's really powerful.”

## Discussion

The results from this research reveal the strengths and limitations of XR technology as it applies to sport. The XR programmers highlighted the key limitations of the XR software and hardware (e.g. limited frame rate/refresh rate of VR headsets, lack of haptic feedback) which they believed would significantly reduce the extent to which XR can represent the perception–action couplings that exist during real-world performance. This notion has been supported recently by Müller and colleagues^24^ who suggested that misrepresenting visual information within VR environments can have a significant effect on athletes’ performance, including drawing their attention to later occurring visual information which causes their responses to be late or rushed, compared to when performing in the real-world environment [24]. This is likely to reduce the probability of positive skill transfer occurring from the XR setting to the real-world environment [5, 10]. Indeed, this has been an overarching issue surrounding the use of sport-specific XR tools in the past [11]. Training with XR tools that misrepresent the actions performed in the real-world setting is potentially detrimental for athletes’ skill development and could lead to undesirable movement patterns, or a worsening of performance [9]. Therefore, sports practitioners should be cautious when utilising animated VR for training motor skills [5].

This result highlights that the XR programmers’ thoughts were in line with skill acquisition principles such as those outlined in the theory of representative learning design [10] which provides a framework for guiding the design of XR training tools [5]. Representative learning design suggests that XR training tools require both functionality and action fidelity to maximise their effectiveness [5, 10]. Functionality refers to athletes basing their decision-making and actions on comparable information to that of the real performance environment [9]. Action fidelity refers to whether athlete behaviour remains the same in the training and performance environment [25, 26]. Therefore, it is essential that XR training tools sample information found in specific performance settings and that those tools also ensure the functional coupling of perception and action [5, 10]. Doing so allows athletes to regulate their movement behaviours based upon information found in the performance setting, thus enhancing the likelihood of skill transfer occurring [10, 25].

The XR programmers also revealed a number of practical applications for XR technology in sport that have potential for enhancing athlete and coaching performance and also for improving the current methods used to educate or train players’ perceptual–cognitive skill and performance under pressure. The opportunities to store and measure athlete performance when using XR tools for these types of training interventions were also demonstrated. The XR programmers’ opinions are in line with previous research and reviews in the area of XR technology in sport, which suggest that the use of animated VR for tactical decision-making and for pressure exposure training are fruitful areas of development [5, 27]. Indeed, a recent study that trained the tactical decision-making of basketball players using animated VR found promising results [27]. Players were immersed in an animated VR basketball environment where they learnt how to perform tactics by interacting with virtual players and a virtual basketball from a first-person viewing perspective. The VR training group showed significant improvements in their movement patterns (correctness of running position) from pre- to post-test when implementing complex tactics, compared to groups that trained using a whiteboard or 2D video screen who showed no improvement. Additionally, in other domains including the military and psychology, anxiety levels and post-traumatic stress disorder have both been effectively treated using animated VR exposure therapy techniques [1, 28, 29]. Therefore, the results of these studies provide further support for utilising animated VR for these types of training interventions in sport.

In-line with previous suggestions from Le Noury and colleagues^5^, the use of AI for training athletes’ tactical decision-making was supported by the XR programmers. This type of tactical decision-making training can overcome some of the challenges of perceptual–cognitive skill interventions that have relied upon 2D images. Such interventions have been limited by an inability to interact with stimuli and the fact that the 2D images are pre-recorded and therefore difficult to further manipulate. Notably, the use of AI for enhancing coaches’ decision-making during live competition was a key suggestion made by the XR programmers. This idea has also been suggested in a review paper focused on the use of AI for enhancing player and coaching performance in soccer [30].

In addition, the XR programmers discussed the novel idea of using AI to help athletes develop and create new movement solutions in specific sporting contexts. The development of new movement techniques can be extremely challenging in many sports (e.g. BMX freestyle, skateboarding) and is limited by the creativity of the athletes and coaches. The programmers suggested that AI has high potential to help overcome this issue by accelerating the rate at which new movement techniques can be created to extend those that have previously been performed in competition. Moreover, it was noted that animated VR can be used as a training tool to facilitate the capability of the athletes to learn the new movement techniques that the AI generates. For example, athletes could observe and analyse the new techniques that the AI agent is performing whilst immersed in an interactive animated VR environment. Athletes could zoom in and/or freeze the AI agent’s technique to observe different angles or to explore different features of the movement.

## Conclusions

This is the first time that XR programmers have been interviewed about the use of this technology in high-performance sport. The responses provided by the XR programmers help to confirm the results of other research by further highlighting the limitations of XR technology and the issues associated with creating representative simulations of real-world sporting environments. The results provide sporting organisations, coaches, athletes, and XR technology companies with insights into where XR technology can have the greatest positive impact on performance in sport before they invest large amounts of time, effort, and money into XR technology. The results also highlight the limitations of XR technology, as well as potential research directions for further exploring the utility of XR into the future. These research areas may include whether factors such as the reduced framerate, the change in physical properties, and the absence of haptic feedback in the XR setting have an impact on the learning of perceptual–motor skills. Additional areas worth exploring further include whether VR technology can be used for exposure therapy to prepare athletes for competition and to provide coaches with live feedback during competitions. Overall, the use of XR technology in sport is in its infancy and more research is warranted to establish a deeper understanding of its utility and effectiveness.

## Data Availability

The data sets generated and/or analysed during the current study are not publicly available due to the risk of exposing the identity of participants.

## Appendix

### Interview questions guide

The interview questions guide included the following:

Section 1: Warm-up questions

1. What is your current role at this company and what projects are you currently working on?
2. What is your current knowledge and experiences of the sport industry?

Section 2: Representativeness and utilisation of XR in sport

1. Do you think XR can reproduce a realistic sports environment?
2. Sports coaches and researchers use many measures to assess the performance of athletes. What types of performance measures are able to be collected in the XR environment?
3. If you were employed as the XR programmer at an elite sporting organisation, how would you use XR to improve the performance of your athletes?
4. What does the future of XR in sport look like?

## Abbreviations

AI: Artificial intelligence
AR: Augmented reality
XR: Extended reality
VR: Virtual reality
MR: Mixed reality
